# Expanding access to NCD services via community retail pharmacies in LMICs: a systematic review of the literature

**DOI:** 10.1080/20523211.2025.2462450

**Published:** 2025-02-17

**Authors:** Arianna Gentilini, Lombe Kasonde, Zaheer-Ud-Din Babar

**Affiliations:** a Department of Health Policy, London School of Economics and Political Science, London, UK; b Health, Nutrition and Population Global Practice, World Bank Group, Washington, DC, USA; c Department of Clinical Pharmacy and Practice, College of Pharmacy, QU Health, Qatar University, Doha, Qatar

**Keywords:** Non-communicable diseases, community pharmacies, low and middle-income countries, package of essential non-communicable disease interventions

## Abstract

**Background:**

Non-communicable diseases (NCDs) pose a significant global health challenge. In LMICs, NCDs are an incresing driver of premature deaths and have substantial economic impacts, particularly on working-age adults. The World Health Organization has identified four priority NCDs – cardiovascular diseases, diabetes, asthma/chronic obstructive pulmonary disease, and cancer – which are included in its Package of Essential Non-Communicable Disease Interventions for low-resource primary care settings. However, a shortage of healthcare professionals further compounds the problem. Pharmacists, who could play a pivotal role in NCD care, remain underutilised.

**Methods:**

We conducted a systematic literature review to identify studies on the role of community pharmacies and pharmacists in delivering NCD services in low- and middle-income settings and assessed their risk of bias. Searches were performed in PubMed, MEDLINE via Ovid, and CINAHL from 1990 to 2022, including English, French, or Spanish publications.

**Results:**

Out of 1,284 articles, 23 met inclusion criteria, predominantly focusing on diabetes (65%), followed by cardiovascular diseases (22%), cancer (9%), and asthma (4%). Most studies were conducted in Asia (52%), followed by South America (22%) and Africa (13%). Significant improvements were observed in glycaemic control and medication adherence for diabetes, and in blood pressure management and adherence for hypertension. Positive outcomes were also seen in health behaviours for breast cancer, asthma, and cardiovascular disease risk management. Interventions were cost-effective for managing diabetes and hypertension in elderly patients. However, accessibility challenges were noted in vaccination programmes, and concerns about bias were identified, particularly in observational studies.

**Conclusions:**

Expanding NCD services through community pharmacies in low and middle-income countries can significantly improve health outcomes. Pharmacists can enhance education, screening, and management for NCDs, leading to better disease control and patient satisfaction. Addressing resource constraints, legal barriers, and disease focus disparities is essential. Adequate training, financial incentives, and collaboration among stakeholders are crucial for integrating pharmacists into NCD care frameworks.

## Background

Non-communicable diseases (NCDs) pose a significant global health concern, causing over 36 million deaths each year (World Health Organization, 2020b). NCDs include cardiovascular diseases, cancer, chronic respiratory diseases, and diabetes, and are linked to modifiable risk factors such as smoking, excessive alcohol consumption, lack of physical activity, and unhealthy diets (Akutey et al., 2018; World Health Organization, 2023a). Tackling NCDs is critical to achieving the United Nations’ Sustainable Development Goal 3, which aims to reduce premature NCD mortality by a third by 2030 (Akutey et al., 2018; Mutale et al., 2018). While NCDs are a challenge in high-income countries and account for 75% of non-pandemic-related mortality globally, they are even more burdensome in low and middle-income countries (LMICs), where they contribute to approximately 80% of premature NCD-related deaths (World Health Organization, 2023a). The economic impact of NCDs is also substantial, as they disproportionately affect working-age adults, leaving nations without an economically productive population (Gheorghe et al., 2018; Nyarko et al., 2016). LMICs require around $11.4 billion annually to implement effective NCD prevention and treatment strategies (Bloom et al., 2011). This, combined with the high risk of catastrophic expenditure associated with NCDs, make it imperative that domestic resources are mobilised and national and global plans are developed and implemented to tackle this growing threat (Kazibwe et al., 2021).

The prevention and control of NCDs and their risk factors in LMICs require cost-effective, affordable, and feasible interventions based on local resources (Pinto et al., 2020). The World Health Organization (WHO) developed the Package of Essential Non-Communicable Disease Interventions (PEN) to support responses to NCDs in low-resource primary care settings, referred to herein as WHO PEN (World Health Organization, 2023b). WHO PEN provides clinical decision support for the assessment and management of NCDs and focuses on four priority areas: cardiovascular diseases, diabetes, asthma/chronic obstructive pulmonary disease (COPD), and cancer (World Health Organization, 2023b). For cardiovascular disease and stroke, it promotes lifestyle changes like tobacco cessation and salt reduction alongside medications – such as aspirin, statins, and antihypertensives – for high-risk individuals. In diabetes care, type 1 patients receive insulin injections, while type 2 patients start with lifestyle changes and, if needed, oral hypoglycemic drugs. Cardiovascular risk reduction is addressed through aspirin, ACE inhibitors, and statins. Preventive foot, kidney, and eye exams help manage common diabetes complications like ulcers, kidney damage, and vision loss. For asthma and COPD, the focus is on symptom relief through short-acting bronchodilators and inhaled corticosteroids, along with smoking cessation support to prevent disease progression. Finally, cancer management in WHO PEN emphasises early detection and referral for suspected cases of common cancers, enabling timely diagnosis and treatment when resources allow. The WHO PEN intervention set is intended to serve as a minimum standard for the management of NCDs in low-resource settings, increasing the national capacity to integrate and improve primary care interventions for the major NCDs. These interventions can be delivered by primary care physicians and non-physician health workers, such as pharmacists (World Health Organization, 2023b; Zhang et al., 2015).

The implementation of WHO PEN has been tested in various countries globally, including in South and Central Asia (Tripathy & Mishra, 2021). Although studies have shown that the PEN interventions are cost-effective, several LMICs have reported difficulties with poor availability of manpower, essential medicines, equipment, and other supplies at primary care facilities (Nyarko et al., 2016; Tripathy & Mishra, 2021). This lack of resources has been identified as a major barrier to the success of WHO PEN and highlights the need for adequate staffing and provision of essential resources, including fair financing, trained personnel, equipment, diagnostics, and medications. To address these challenges, scholars have highlighted the need to empower non-physician health workers to deliver NCD interventions in line with their level of care (Nyarko et al., 2016; Pinto et al., 2020).

Pharmacists have the potential to play a crucial role in the global fight against NCDs. As the first point of contact for most patients seeking healthcare, pharmacists can be a valuable part of the multidisciplinary healthcare team, conducting screening and referrals, management, and prevention of diseases (Babar, 2021; Pinto et al., 2020). This is particularly relevant in LMICs, where access to health facilities and health workers providing services of adequate quality are limited (Akutey et al., 2018). The scarcity of hospitals and physicians in rural areas presents an opportunity to make use of community pharmacies as a key source of integrated NCD services for patients (Mutale et al., 2018). Additionally, with the use of new technologies and their expertise, pharmacists can be critical to improving patient adherence to safe and effective therapeutic plans, ultimately improving quality of life and reducing healthcare costs (Akutey et al., 2018; Pinto et al., 2020). The ongoing revision and drafting of NCD strategies in several LMICs presents a unique opportunity for these countries to re-evaluate the role of pharmacists and pharmaceutical services in addressing the NCD burden (World Health Organization, 2020a).

This systematic review aims to understand the role of community pharmacists and pharmacy services in supporting the delivery of interventions included in WHO PEN in LMICs. The research question of this review article is whether pharmaceutical services are an effective, efficient, and accessible entry point to support the delivery of NCD interventions. We respond to this question by conducting a systematic literature review to identify studies on pharmaceutical services for NCDs in low- and middle-income settings. We also assessed the included study to identify barriers to pharmacists’ involvement in disease prevention and management. Specifically, we examine whether the legal framework restricts pharmacists from expanding their role or if other healthcare professionals oppose enhanced pharmacists’ responsibilities in disease prevention and monitoring.

The paper is structured as follows. The Methods section describes the methodology for the literature review, with results presented in the Results section. Finally, the Discussion section concludes and discusses policy implications.

## Methods

We conducted a systematic literature review to identify studies on the role of community pharmacies and pharmacists in delivering NCD services in low- and middle-income settings. This study follows the Preferred Reporting Items for Systematic Reviews and Meta-Analyses (PRISMA) (Moher et al., 2009). To address our research question, we also defined effectiveness, efficiency, and accessibility, of health services.

### Effectiveness

Effectiveness in healthcare refers to the degree to which health services achieve health improvements in real-world settings (Cullen & Ergas, 2017). It answers questions such as whether an intervention works, and if it causes more harm than good. In this study, pharmaceutical services are considered effective if they produce the intended health outcomes for their target patients. For example, a pharmacy-based blood sugar monitoring team would be considered effective if it can identify patients who require further care and direct them to the appropriate treatment or help people manage their diet successfully.

### Efficiency

Efficiency in healthcare refers to the optimal use of resources to achieve the best value for money. It is concerned with the relationship between inputs (such as resources, time, and money) and outputs (performance and outcomes). An efficient health service achieves a high level of performance relative to the resources consumed (Palmer & Torgerson, 1999). In the context of pharmaceutical services, efficiency means that they cannot be improved without incurring additional costs. For instance, a pharmacist working 6 hours and assisting only one patient could be considered inefficient, as they could potentially assist more patients without being contracted to work longer hours.

### Accessibility

According to the WHO, accessibility in healthcare refers to the availability and ease of use of health services for all people and communities, without financial burden (World Health Organization, 2023c). In the context of pharmaceutical services, accessibility means that there are no substantial barriers preventing patients from benefiting from these services. This includes issues such as travel time or cost, for example.

### Data collection and extraction

We developed a search strategy in PubMed building on previous reviews (Ayorinde et al., 2013; Maria et al., 2020), which we modified for use in other databases (MEDLINE via Ovid and CINAHL) to identify relevant articles from 1990 to 2022. This time restriction was applied to exclude older studies that may no longer be relevant because of changes in treatment, policy, or disease burden. The databases were searched for a combination of terms relating to pharmaceutical services (e.g. ‘pharmacies’, ‘community pharmacies’, ‘pharmacists’, ‘pharmaceutical services’, ‘pharmacy assistant’, ‘pharmacy technician’, ‘counter assistant’) and NCDs (e.g. ‘NCD’, ‘non-communicable diseases’, ‘cardiovascular diseases’, ‘CVD’, ‘diabetes’, ‘respiratory diseases’, ‘COPD’, ‘asthma’, ‘cervical cancer’, ‘breast cancer’, ‘cancer screening’, ‘tobacco cessation’). Our search strategy also included all low- and middle-income countries as defined by the World Bank (Maria et al., 2020). The full search strategy for the different databases can be found in the Appendix (Tables A1–A3).

Results were restricted to publications in English, French or Spanish. These languages were selected to minimise language-based restrictions while aligning with the authors’ language proficiency. Eligible studies were peer-reviewed original articles, analyses, literature reviews, and meta-analyses that addressed the delivery of NCD services within community pharmacy settings in LMICs, such as screening, prevention, management, pharmaceutical care, referral, and follow-up. No specific exclusions were applied based on the study’s methodological approach. Table 1 summarises the inclusion and exclusion criteria for the analysis.

**Table 1.** Inclusion and exclusion criteria.

Category	Inclusion Criteria	Exclusion Criteria
Language of publication	English, French, Spanish	Published in any other language
Year of publication	January 1990–December 2022	Published before 1990
Geographic scope	low- and middle-income countries as defined by the World Bank	Studies not including low- and middle-income countries
Study type	Peer-reviewed original articles, analyses, literature reviews, meta-analyses	Non-peer-reviewed literature (e.g. news, reports), commentaries, editorials, conference abstracts (i.e. no full-text available), clinical studies
Outcomes	Studies included must discuss delivery of NCD services at the pharmacy level in LMICs (e.g. screening, prevention, management, pharmaceutical care, referral, follow-up)	– Studies discussing delivery of NCD services in non-pharmacy settings (e.g. hospital) – Studies on how pharmaceutical services can be deployed to target any disease other than NCDs (e.g. HIV, malaria) – Studies targeting telemedicine interventions
Methodology	Observational studies, surveys, interviews, and more	No methodology constraint was applied

Abbreviations: HIV, human immunodeficiency virus; LMICs, low- and middle-income countries; NCD, non-communicable diseases.

Identified publications were then streamlined through a two-stage exclusion-inclusion process. Initially, publication titles and abstracts were reviewed and decisions to exclude were made on the basis of their relevance. Records that were included based on the first screening, were re-assessed on the basis of their full-text. During the full-text review stage, reference lists of the studies were manually screened to identify publications that might meet the study inclusion criteria. Publications that passed both screening steps were included for extraction. Key information extracted included record information, author affiliation, geographic scope, and considerations around accessibility, efficiency and effectiveness.

### Risk of bias assessment

Subsequently, the quality and the risk of bias (ROB) of the included studies were evaluated using validated tools according to their study design. As recommended by the Cochrane Collaboration, for randomised controlled trials (RCTs), we used the RoB 2 tool, which evaluates bias across five key domains: bias arising from the randomisation process (D1), bias due to deviations from the intended interventions (D2), bias due to missing outcome data (D3), bias in measurement of the outcome (D4), bias in selection of the reported result (D5). For observational studies, we used the ROBINS-I tool, which evaluates bias across a broader set of domains to capture specific non-randomised study factors. These domains include some discussed above (D2-5), well as the following: bias due to confounding (D6), bias in selection of participants into the study (D7), bias in classification of interventions (D8). An overall bias (OB) score summarises the cumulative risk of bias for each of the study assessed (Sterne et al., 2016, 2019).

## Results

**Figure 1.**
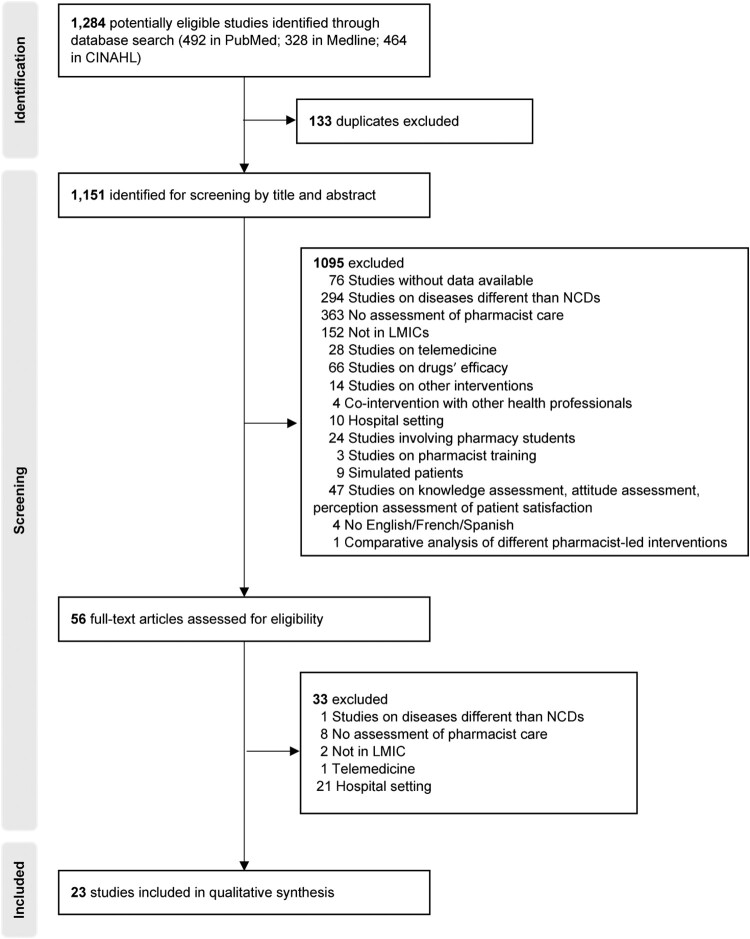
PRISMA flow diagram.

In total, 1,284 articles were identified initially from three electronic databases, resulting in 1,151 unique citations. After performing title and abstract screening, 56 full-text were assessed for eligibility, of which 23 articles met the inclusion criteria. A flow chart summarising the selection procedure is shown in the PRISMA flow diagram of Figure 1.

One researcher (AG) independently collected data from each identified study, recording it on an Excel-based data extraction form. To ensure its effectiveness in capturing all relevant information, the data extraction form was pilot-tested using a selection of included studies. The extracted data from these studies encompassed various details, such as author names, author affiliations, year of publication, journal, year of data collection, population of interest, country, NCD(s), study objective, primary and secondary outcomes, study setting, intervention, comparator, study design, duration, sample size, and key results. Key study characteristics and results can be found in Table 2.

### Study characteristics

Among the 23 included studies, most focused on diabetes (n = 15, 65%), followed by cardiovascular diseases (n = 5, 22%), cancer (n = 2, 9%), and asthma (n = 1, 4%). In terms of geographic location, most of the studies were conducted in Asia (n = 12, 52%), followed by South America (n = 5, 22%) and Africa (n = 3, 13%), with the remaining three studies being literature reviews covering multiple geographical regions (13%). The pharmacist-led interventions took place in community pharmacies or primary healthcare facilities. The study designs of the studies that met the inclusion criteria included RCTs (n = 12, 52%), observational trials (n = 8, 35%), and systematic literature reviews (n = 3, 13%). The intervention duration spanned from 1 month to 3 years. The interventions consisted of educational and counselling sessions led by the pharmacist(s), mostly targeting key outcomes such as medication adherence, disease knowledge, self-management skills, and objective measures like glycaemic levels, blood pressure, lipid profiles, and health-related quality of life scores. One study focused on assessing the cost-effectiveness of pharmaceutical care, while another study aimed at assessing the feasibility of implementing a pharmacy-based CVD risk screening service in a referral pharmacy (Jahangard-Rafsanjani et al., 2017; Obreli-Neto et al., 2015).

### Effectiveness, efficiency, and accessibility of pharmaceutical services

Overall, the results showed positive outcomes, particularly in effectiveness, with improvements in medication adherence, disease state knowledge, and patient satisfaction. Evidence demonstrated that pharmacist-led interventions led to significant improvements in glycaemic control and medication adherence among diabetes patients (Abubakar & Atif, 2021; Abubakar et al., 2021; Alfian et al., 2021; Bello et al., 2014; Besemah et al., 2020; Bukhsh et al., 2018; Javaid et al., 2019; Mourão et al., 2013; Presley et al., 2019; Rosli et al., 2021; Venkatesan et al., 2012). Improvements in glycaemic control were reported by several studies. For example, Abubakar and Atif (2021) found significant improvements in fasting and random blood glucose in the pharmacist intervention arm. Similarly, Javaid et al. (2019) demonstrated significant reductions in HbA1c, systolic blood pressure, diastolic blood pressure, cholesterol, and triglycerides.

Pharmacist-led interventions also positively impacted hypertension management. Aguiar et al. (2012) reported significant reductions in systolic and diastolic blood pressure and improved medication adherence among elderly patients. Marfo and Owusu-Daaku (2017) observed significant reductions in diastolic blood pressure and improved adherence among hypertensive patients. In addition to improvements in diabetes and hypertension management, pharmacist-led interventions improved health behaviours related to breast cancer (Ibrahim et al., 2021), asthma control (Wong et al., 2017), and cardiovascular disease risk management (Jahangard-Rafsanjani et al., 2017).

Only two studies addressed efficiency concerns (Jiménez-Quiñones et al., 2017; Obreli-Neto et al., 2015), and one assessed accessibility (Jiménez-Quiñones et al., 2017). More specifically, one study evaluated the cost and incremental cost-effectiveness ratio per quality-adjusted life-year of pharmaceutical care in managing diabetes and hypertension in elderly patients in Brazil (Obreli-Neto et al., 2015). The study found that pharmaceutical care did not significantly increase total direct healthcare costs, while significantly improving health outcomes, suggesting that pharmaceutical care is a cost-effective intervention. Another study evaluating the role of pharmacists in human papillomavirus (HPV) vaccinations in Brazil indicated that reaching patients was challenging, with attendance being very low even after appointments were made due to working commitments and low-risk perception (Jiménez-Quiñones et al., 2017).

### Legal framework

Issues with the existing legal framework were raised in two studies, affecting the accessibility of services. A study in Puerto Rico assessing whether local vaccination rates are improved by HPV education programmes found that legal barriers prevent pharmacists from vaccinating patients younger than 18 years old, despite medical recommendations for HPV vaccinations in patients from 9 to 26 years old (Jiménez-Quiñones et al., 2017). In another study set in Ghana, it was noted that pharmacists are limited in their actions towards treating and managing hypertensive patients due to the Health Professions Regulatory Bodies Act 867 not making clear which are the responsibilities of community pharmacists for patients with chronic diseases (Marfo & Owusu-Daaku, 2017) .

**Table 2.** Details of included studies.

Author, Year	Country	NCD(s)	Study design	Duration of study (in months)	Sample size	Primary outcome*	Study goal	Results
(Abubakar & Atif, 2021)	Pakistan	T2DM	RCT	1 month	160	Glycemic control fasting blood glucose, random blood glucose, medication adherence, health related quality of life	To determine the impact of pharmacist-led interventions on glycemic levels, medication adherence, and quality of life for Pakistani people with diabetes attending a community pharmacy-based service	Pharmacist intervention significantly improved fasting blood glucose and random blood glucose. Medication adherence and HRQoL also showed clinically significant improvements
(Abubakar et al., 2021)	Pakistan	T2DM	Quasi-experimental study	1 month	80	Satisfaction, disease state knowledge, perception of self-management	To determine the impact of pharmacist-led interventions on satisfaction, disease state knowledge and perception of self-management of diabetes patients	Significant improvements in patient satisfaction, disease state knowledge, and self-management of diabetes post-intervention
(Aguiar et al., 2012)	Brazil	Hypertension	Non-randomised, single-intervention, pre/post-test	10 months	35	Target blood pressure control, reduction in blood pressure, pulse pressure, medication adherence, reduction of anthropometric indices	To evaluate the effect of a pilot pharmaceutical care program developed for elderly patients with uncontrolled hypertension	57.2% of elderly patients achieved blood pressure control; significant reductions in systolic, diastolic, and pulse pressure. Improved medication adherence (*P* = 0.000)
(Alfian et al., 2021)	Indonesia	T2DM	Cluster-RCT	3 months	89	Medication adherence	To assess the effects of a targeted and tailored pharmacist-led intervention among patients with T2DM who are nonadherent to antihypertensive drugs	Medication adherence improved by 4.62 points on the MARS-5 scale (95% CI 0.93–8.34, *P* = 0.008). No significant changes in blood pressure or beliefs about antihypertensive drugs
(Bello et al., 2014)	Nigeria	T2DM	Observational study, pre/post-test	3 months	170	Changes in fasting blood sugar, glycosylated hemoglobin, body mass index, blood pressure	To evaluate pharmacists’ intervention in the control of blood sugar levels of diabetes patients in a PHC setting in Benin City, Nigeria	Significant reductions in BMI (from 27.1 ± 4.2–23.5 ± 3.5 kg/m², *P* < 0.001), HbA1c (from 8.1 ± 3.0% to 7.1 ± 1.8%, *P* < 0.001), and fasting blood sugar (from 10.0 ± 4.2–8.5 ± 2.1 mmol/L, *P* < 0.001)
(Besemah et al., 2020)	Indonesia	T2DM	Prospective quasi-experimental nonrandomised control study with pre/post-test	4 months	80	Glycated hemoglobin, blood pressure, lipid profiles	To evaluate the effectiveness of programmes delivered for T2DM patients by pharmacists in primary healthcare through counseling, short message service reminders, and medication booklets	Intervention group showed significant improvements in HbA1c, TC, LDL c, TG, and medication adherence. Control group showed decreased medication adherence and worsening clinical outcomes
(Bukhsh et al., 2018)	Asia (*n* = 5), Europe (*n* = 2), North America (*n* = 4)	T2DM	Systematic literature review and meta-analysis	4–12 months	1,544	Glycemic control, diet control, physical activity, self-monitoring of blood glucose	To appraise the effect of pharmacist-led educational interventions on self-care activities and levels of glycated hemoglobin of T2DM patients	Meta-analysis showed significant reduction in glycated hemoglobin. Significant improvements in self-monitoring of blood glucose, foot care, and overall diet
(Fajriansyah et al., 2020)	Indonesia	T2DM	Cluster-RCT with pre/post-test	6 months	220	Quality of life	To analyse the impact of pharmacist counseling interventions on HRQoL in T2DM patients enrolled in the program of management of chronic diseases ‘Program Pengelolaan Penyakit Kronis’ (Prolanis)	Significant improvement in EQ-5D-5L index score (0.04 vs 0.01, *P* = 0.041) and VAS score (2.66 vs – 0.07, *P* = 0.000) in the intervention group
(Ibrahim et al., 2021)	Egypt	Breast cancer	RCT	3 months	232	Health behaviours, breast-cancer knowledge	To investigate the effectiveness of pharmacist-based coaching in improving BC-related health behaviours and knowledge in females, and to measure the comfort level toward this program	Post-coaching, higher proportions of physical activity (52.17% vs 17.09%, *P* = 0.002), healthy diet (62.60% vs 28.20%, *P* = 0.003), and breast self-exam practice (81.73% vs 23.07%, *P* = 0.005) in the intervention group. Improved BC knowledge scores. Some discomfort with coach competency
(Ifeanyi Chiazor et al., 2015)	Africa (*n* = 2), Australia (*n* = 3), Europe (*n* = 9), North America (*n* = 10), South America (*n* = 3)	CVD	Systematic literature review	3–24 months	27	Cardiovascular risk factors	To assess the effectiveness of community pharmacists’ interventions in reducing major risk factors for cardiovascular diseases	Significant reductions in systolic blood pressure (7.8–17.7 mm Hg) and HbA1c (0.2% to 2.2%). Reductions in total cholesterol (18.2–27.1 mg/dL). The quality of included studies was generally poor
(Jahangard-Rafsanjani et al., 2017)	Iran	CVD	Cross sectional study	1 month	287	Assessment of CVD risk profile of participants, number of referrals by community pharmacist	To assess the outcomes and feasibility of a pharmacy-based cardiovascular screening in an urban referral community pharmacy in Iran	Identified high CVD risk; follow-up led to physician appointments and appropriate medication/advice for 15.9% of individuals
(Javaid et al., 2019)	Pakistan	T2DM	Open-label RCT	9 months	244	A1C hemoglobin, blood pressure, lipid controls	To demonstrate the pharmacist-led improvements in glycemic, blood pressure and lipid controls in T2DM patients of Lahore, Pakistan	Significant improvements in HbA1c, systolic, diastolic BP, cholesterol, and triglycerides
(Jiménez-Quiñones et al., 2017)	Puerto Rico	Cervical cancer	Prospective descriptive study	N/A	79	HPV vaccination rates	To observe whether local vaccination rates are improved by a patient and physician education program on the HPV vaccine in Farmacia San José, Lares	Limited intervention reach; four patients received HPV vaccination following educational efforts
(Malik et al., 2022)	Pakistan	Diabetes (Type I or II), hypertension	Randomised, controlled, single-blind, pre-post-intervention study	6 months	80	Blood pressure, blood glucose	To evaluate the impact of pharmacist counseling on blood pressure and blood glucose control among patients having both hypertension and diabetes attending community pharmacies in Pakistan	Significant improvements in knowledge scores for diabetes (16.02 ± 2.93–19.97 ± 2.66) and hypertension (15.60 ± 3.33–18.35 ± 2.31) (*P* < 0.05), fasting blood glucose (8.25 ± 1.45), and systolic BP (130.10 ± 6.89)
(Marfo & Owusu-Daaku, 2017)	Ghana	Hypertension	Quasi-experimental study	6 months	146	Systolic and diastolic blood pressure	To evaluate the effect of a pharmaceutical care model on blood pressure control and adherence among hypertensive patients	Significant reductions in diastolic blood pressure (*P* = 0.001) and improved adherence (*P* = 0.001) in the intervention group
(Mourão et al., 2013)	Brazil	T2DM	Open RCT	6 months	100	Serum level of haemoglobin A1C	To evaluate the effect of a pharmaceutical care program on blood glucose, blood pressure and lipid profile in hyperglycaemic patients undergoing drug treatment for type 2 diabetes	Significant reductions in HbA1c (−0.6 vs 0.7%, *P* = 0.001), fasting plasma glucose, total and LDL cholesterol, triglycerides, and systolic blood pressure; increased HDL cholesterol
(Obreli-Neto et al., 2011)	Brazil	Diabetes (Type I or II), hypertension	Randomised, controlled prospective clinical trial	36 months	194	Pharmacotherapy adherence, blood pressure, fasting glucose, A1C hemoglobin, triglycerides, total cholesterol	To evaluate the effect of a pharmaceutical care program on pharmacotherapy adherence in elderly diabetic and hypertensive patients	Significant improvements in adherence, blood pressure, fasting glucose, HbA1c, triglycerides, and total cholesterol in the intervention group
(Obreli-Neto et al., 2015)	Brazil	Diabetes (Type I or II), hypertension	Randomised, controlled longitudinal clinical trial	36 months	194	Direct costs of health services, ICER per QALY	To evaluate the economic cost and the ICER per QALY of pharmaceutical care in the management of diabetes and hypertension in elderly patients in a primary public health care system in a developing country	No significant difference in total direct health care costs ($281.97 ± $49.73 vs. $212.28 ± $43.49, *P* = 0.089); ICER per QALY of $53.50 (95% CI = $51.60-$54.00)
(Pongwecharak & Treeranurat, 2011)	Thailand	CVD	Descriptive, exploratory, nonexperimental pre/post-test	3 months	400	Number of prehypertensive/hypertensive participants, patient return rate, rate of laboratory referral uptake, confirmed glucose intolerance and dyslipidemia, blood pressure level	To evaluate a model for community pharmacists to screen and recommend lifestyle changes for patients with prehypertension/ hypertension and other elevated modifiable cardiovascular risk factors	Significant reductions in systolic (6.5 ± 0.89 mm Hg, *P* < 0.001) and diastolic BP (2.2 ± 0.82 mm Hg, *P* = 0.009); increased normotensive participants (39.3% to 51.8%, *P* < 0.001); lifestyle changes reported but no change in smoking and physical exercise
(Presley et al., 2019)	Africa (*n* = 2), Asia (*n* = 27), Australia (*n* = 2), Europe (*n* = 7), North America (*n* = 17), South America (*n* = 4)	Diabetes (Type I or II)	Systematic literature review	N/A	59	Medication adherence, A1C hemoglobin, fasting plasma glucose, post-prandial blood glucose, random blood glucose	To review pharmacist-led interventions to improve medication adherence in patients with diabetes and to assess the effectiveness of these interventions on medication adherence	Pharmacist-led interventions significantly enhanced medication adherence (SMD – 0.68; 95% CI – 0.79, – 0.58; *P* < 0.001) and glycemic control
(Rosli et al., 2021)	Malaysia	T2DM	RCT	6 months	166	A1C hemoglobin	To evaluate the programme effectiveness of home medication review by community pharmacists in optimising diabetes care and reducing medication wastage	Significant reductions in HbA1c (−0.91%), FBG (−1.62 mmol/L), DBP, and TC; increased diabetes knowledge and reduced drug-related problems in the intervention group
(Venkatesan et al., 2012)	India	T2DM	RCT	2 months	39	Diabetes Care Profile scores, patients’ knowledge about diabetes and its management	To study the role of the community pharmacists in improving knowledge and glycemic control in patients with T2DM residing in villages of Coimbatore district, Tamil Nadu	Significant improvements in Diabetes Care Profile subscale scores and knowledge test scores in the intervention group
(Wong et al., 2017)	Malaysia	Asthma	Cluster RCT	3 months	157	Asthma control, inhaler technique using, medication adherence	To assess the effects of a Pharmacy Management Service on asthma control of adult patients	Significant improvement in asthma control (90% vs. 28.6%, *P* < 0.001), inhaler technique, and medication adherence (92.5% vs. 45.5%, *P* < 0.001) in the intervention group

Abbreviations: BC, breast cancer; BMI, body mass index; CVD, cardiovascular disease; EQ-VAS, EuroQol visual analogue scale; FBG, fasting blood glucose; HbA1c, glycated hemoglobin; HPV, human papilloma virus; HRQoL, health-related quality of life; ICER, incremental cost-effectiveness ratio; LDL, low-density lipoprotein; MARS-5, medication adherence report scale-5; PHC, primary health care; QALY, quality-adjusted life-year; RBG, random blood glucose; RCT, randomised controlled trial; T2DM, type II diabetes mellitus; TC, total cholesterol; TG, triglycerides; VAS, visual analogue scale.

### Risk of bias assessment

Out of the 12 RCTs, 11 (92%) had quality concerns, with 1 (8%) considered at a high risk of bias. Among the 8 observational studies, 3 (37.5%) were assessed as having a moderate risk of bias, while 5 (62.5%) had a serious risk of bias. The majority of RCTs demonstrated appropriate randomisation and clear reporting of results as per the analysis plan and methods. Most observational studies accurately classified intervention status and measured outcomes appropriately. However, there was insufficient explanation of missing data in the majority of studies. Results of the ROB assessment are presented in Tables 3 and 4.

**Table 3.** RoB 2 assessment.

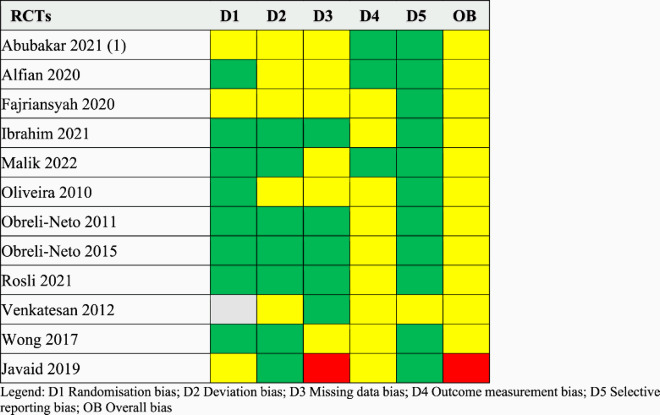

**Table 4.** ROBINS I assessment.

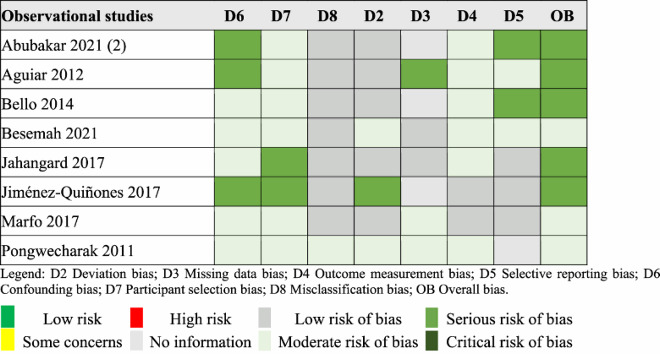

## Discussion

NCDs present a pressing global health priority, and their impact is expected to escalate further in LMICs (GBD Causes of Death Collaborators, 2017; Prüss-Ustün et al., 2019). Community pharmacies can play a vital role in NCD prevention and management, particularly in underserved regions, where they act as essential access points for healthcare (Akutey et al., 2018; Pinto et al., 2020). Pharmacists not only can provide education on NCDs, but also facilitate screening, prevention activities, and referrals for proper diagnosis and care, empowering patients to manage their conditions effectively (Akutey et al., 2018; Mutale et al., 2018; Pinto et al., 2020).

However, despite their potential, pharmacists remain underutilised in the fight against NCDs. Our findings show that community pharmacists have great potential in providing NCD care and improving health outcomes in LMICs, aligning with existing evidence (Pinto et al., 2020; Rajan et al., 2024). The findings suggest that pharmacist-led interventions yield positive outcomes, particularly in effectiveness, by enhancing medication adherence, disease knowledge, and patient satisfaction. Key improvements were observed in diabetes and hypertension management, with studies reporting significant gains in glycemic control, and health behaviours across various conditions, including asthma, breast cancer, and cardiovascular risk management (Abubakar & Atif, 2021; Abubakar et al., 2021; Alfian et al., 2021; Bello et al., 2014; Besemah et al., 2020; Bukhsh et al., 2018; Ibrahim et al., 2021; Jahangard-Rafsanjani et al., 2017; Javaid et al., 2019; Mourão et al., 2013; Presley et al., 2019; Rosli et al., 2021; Venkatesan et al., 2012; Wong et al., 2017). However, few studies addressed efficiency and accessibility, though those that did suggested pharmacist interventions could be cost-effective without significantly raising healthcare costs (Obreli-Neto et al., 2015). Nonetheless, accessibility issues remain, particularly in preventive care, where challenges such as low attendance due to work and low-risk perception were noted (Jiménez-Quiñones et al., 2017).

Some studies highlighted that despite pharmacists’ potential, legal barriers can significantly impact the scope of services that pharmacists can provide (Jiménez-Quiñones et al., 2017; Marfo & Owusu-Daaku, 2017). Addressing these legal and regulatory issues is crucial for enable the full integration of pharmacists into NCD care frameworks and to address the growing NCD burden (Babar, 2024; Pinto et al., 2020).

A number of potential limitations exist within the current body of literature on the role of pharmacists in NCD care in LMICs. First, there is an uneven focus on NCD interventions, with certain diseases, such as diabetes and cardiovascular diseases, receiving disproportionate attention. While a significant portion of NCD-related deaths can be attributed to these diseases, cancer is the second leading cause of NCD deaths in LMICs (The Global Health Observatory, 2019). Additionally, deaths due to respiratory diseases have surged by one-third since 2000, underscoring their growing relevance combined with pollution concerns (Rylance et al., 2019; The Global Health Observatory, 2019). Second, the potential of community pharmacists in providing NCD services in Africa remains underexplored, with most studies being conducted in Asia and South America. The lack of studies focusing on this region is concerning, especially in light of the fact that the sub-Saharan region is projected to have NCDs as the leading cause of death by 2030 (Nyarko et al., 2016). Third, most studies identified were of medium to low quality, indicating that higher-quality studies are needed to investigate the potential and impact of community pharmacists in LMICs. In our risk of bias assessment, the majority of RCTs had quality concerns, and a significant proportion of observational studies had a serious risk of bias. Robust research methodologies are necessary to draw more reliable and generalisable conclusions. Fourth, the scarcity of evidence regarding the accessibility and efficiency of pharmacist-provided NCDs services in LMICs poses fundamental challenges for large-scale implementation. Understanding cost-effectiveness and ensuring patient can access the interventions they need are crucial steps to successfully adopt effective NCD management practices. Finally, no study specifically assessing the effectiveness or feasibility of administering interventions included in WHO PEN by pharmacists could be found in the literature. Future research should aim to address the abovementioned gaps.

Limitations regarding the study methodology should also be considered. First, the literature review was limited by the scope of the databases searched and the inclusion criteria applied. This may have led to the exclusion of relevant studies published in languages other than English, French, and Spanish, or those not indexed in the selected databases. Additionally, only one author screened the results, which might have led to mistakes.

The results of this study suggest that community pharmacies can be an effective entry point to support the delivery of NCD interventions in low-resources settings and support the call for empowering pharmacists with greater responsibilities in addressing the challenge posed by NCDs in LMICs. Our findings also highlight the need for sustained efforts to address policy gaps and legal obstacles that hinder the realisation of this potential. To achieve this, collaboration with governments, not-for-profit organisations such as the World Health Organisation, the private sector, healthcare professionals, patient associations, and community leaders, is essential. Such collaboration will facilitate the dissemination of best practices, capacity building, and knowledge sharing, promoting the role of pharmacists in NCD care on a broader scale (Babar, 2024).

## Conclusion

Expanding access to NCD services via community pharmacies and pharmacists in LMICs shows significant potential for improving health outcomes. Results of this review suggest that pharmacists could play a critical role in providing education, screening, and management for NCDs, contributing to better disease control and patient satisfaction. However, challenges such as resource constraints, legal barriers, and uneven focus on certain diseases need to be addressed. Stakeholder collaboration is crucial to fully integrating pharmacists into NCD care frameworks and addressing the growing NCD burden effectively.

## Appendix

Search strategy (per database)

**Table A1.** MEDLINE (via Ovid) search strategy.

#	Search strategy
1	Pharmacy services pharmacists’ aides/ or community pharmacy services/ or pharmacy/ or pharmacies/ or pharmaceutical services/ or pharmacist/ or pharmacists/ or (pharmac$ adj3 (shop$ or store$)).tw. or (community adj3 pharmac$).tw. or (pharmac$ adj3 (assistant$ or technician$)).tw.
2	NCDs non communicable disease/ or NCD.mp. or NCDs.mp. or breast cancer/ or cervical cancer/ or cancer screening/ or diabetes mellitus/ or diabetes/ or diabetic/ or chronic obstructive pulmonary disease/ or COPD.mp. or asthma/ or Lifestyle counselling/ or lifestyle counselling/ or tobacco cessation/ or cessation of tobacco or health education/
3	Geographical scope exp developing country/ or exp medically underserved/ or developing countr$.mp. or medically underserved area$1.mp. or low income countr$.mp. or middle income country.mp. or low resource.mp. or resource poor.mp. or global.mp. or exp Africa/ or exp “South and Central America”/ or exp asia/ or exp Caribbean islands/ or exp pacific islands/ or exp eastern Europe/ or exp Indian Ocean/ or south america$1.mp. or Africa$1.mp. or Caribbean.mp. or central America$1.mp. or south America$1.mp. or eastern Europe$1.mp. or pacific island$.mp. or Indian ocean island$.mp. or asia.mp. or Afghan$.mp. or Bangladesh$1.mp. or Benin$.mp. or Burkina Faso.mp. or Burkinabe.mp. or Burundi$.mp. or Cambodia$1.mp. or Central African.mp. or Chad$.mp. or Comor$.mp. or Congo$.mp. or Eritrea$1.mp. or Ethiopia$1.mp. or Gambia$1.mp. or Guinea$1.mp. or Haiti$.mp. or Kenya$1.mp. or Korea$1.mp. or exp North Korea/ or Kyrgyz$.mp. or Liberia$1.mp. or Madagascar.mp. or Malagasy.mp. or Malawi$.mp. or mali$.mp. or mozambi$.mp. or Myanmar$.mp. or Nepal$.mp. or Niger$.mp. or Rwanda$1.mp. or Sierra Leone$.mp. or Somalia$1.mp. or Tajik$.mp. or Tanzania$1.mp. or Togo$.mp. or Uganda$1.mp. or Zimbabwe$.mp. or Angola$1.mp. or Armenia$1.mp. or Beliz$.mp. or Bhutan$.mp. or Bolivia$1.mp. or Cameroon$.mp. or Cape Verde$.mp. or Congo$.mp. or “Côte d'Ivoire”.mp. or Ivory Coast.mp. or Ivorian.mp. or Djibouti.mp. or Egypt$.mp. or El Salvador.mp. or Salvadoran.mp. or Fiji$.mp. or Georgia$1.mp. or Ghana$.mp. or Guatemala$1.mp. or Guyan$.mp. or Hondura$.mp. or Indonesia$1.mp. or India$1.mp. or Iraq$1.mp. or Kiribati.mp. or Kosov$.mp. or Lao$.mp. or Lesotho.mp. or Marshall Islands.mp. or Marshallese.mp. or Mauritania$1.mp. or Micronesia$1.mp. or Moldov$.mp. or Mongolia$1.mp. or Morocc$.mp. or Nicaragua$1.mp. or Nigeria$1.mp. or Pakistan$1.mp. or Papua New Guinea$1.mp. or Paraguay$.mp. or Philippines.mp. or Filipino.mp. or Samoa$1.mp. or sao tome$.mp. or Senegal$.mp. or Solomon Island$.mp. or sri lanka$1.mp. or Sudan$.mp. or Swazi$.mp. or Syria$1.mp. or Timor$.mp. or Tonga$1.mp. or Turkmen$.mp. or Tuvalu$.mp. or Ukrain$.mp. or Uzbek$.mp. or Vanuat$1.mp. or Vietnam$.mp. or West Bank.mp. or Gaza.mp. or Yemen$.mp. or Zambia$1.mp. or Albania$1.mp. or Algeria$1.mp. or “Antigua and Barbuda”.mp. or antiguan.mp. or barbudan.mp. or Argentin$.mp. or Azerbaijan$1.mp. or Belarus$.mp. or Bosnia$1.mp. or Botswana.mp. or Brazil$.mp. or Bulgaria$1.mp. or Chile$.mp. or China.mp. or Chinese.mp. or Colombia$1.mp. or Costa Rica$1.mp. or 44 Cuba$1.mp. or Dominica$1.mp. or Ecuador$.mp. or Gabon$.mp. or Grenad$.mp. or Iran$.mp. or Jamaica$1.mp. or Jordan$.mp. or Kazakhstan$1.mp. or Latvia$1.mp. or Leban$.mp. or Libya$1.mp. or Lithuania$1.mp. or Macedonia$1.mp. or Malaysia$1.mp. or Maldiv$.mp. or mauriti$.mp. or Mexic$.mp. or Montenegr$.mp. or Namibia$1.mp. or Palau$.mp. or Panama$.mp. or Peru$.mp. or Romania$1.mp. or Russia$1.mp. or Serbia$1.mp. or Seychell$.mp. or South Africa$1.mp. or Saint Kitts.mp. or Saint Lucia.mp. or Saint Vincent.mp. or Suriname$1.mp. or Thai$.mp. or Tunisia$1.mp. or Turk$.mp. or Uruguay$.mp. or Venezuala$1.mp.

**Table A2.** PubMed search strategy.

#	Search strategy
1	Pharmacy services “pharmacist” OR “pharmacists” OR “community pharmacists” OR “community pharmacist” OR “pharmacies” OR “pharmacy” OR “community pharmacies” OR “community pharmacy” OR “pharmaceutical services” OR “pharmacy store” OR “pharmacy assistant” OR “pharmacy technician”
2	NCDs “non-communicable diseases” OR “NCDs” OR “NCD” OR “breast cancer” OR “cervical cancer*” OR “cancer screening” OR “diabetes*” OR “diabetic*” OR “chronic respiratory disease*” OR “pulmonary disease” OR “chronic obstructive” OR “chronic obstructive pulmonary disease” OR “copd” OR “asthma*” or “lifestyle counselling” or “lifestyle counseling” or “tobacco cessation” or “cessation of tobacco” or “health education”
3	Geographical scope “developing countries” OR “developing country” OR “developing countries” OR “medically underserved area” OR “medically underserved area” OR “medically underserved areas” OR “low income countries” OR “low income country” OR “middle income countries” OR “middle income country” OR “global” OR “resource poor” OR “low resource” OR “Africa” OR “Asia, Central” OR “Asia, Western” OR “Asia, Southeastern” OR “Indian Ocean Islands” OR “Central America” OR “South America” OR “Europe, Eastern” OR “Transcaucasia” OR “China” OR “Korea” OR “Mongolia” OR “Mexico” OR “Caribbean Region” OR “Pacific Islands” OR “Africa” OR “Central Asia” OR “western Asia” OR “southeastern Asia” OR “Indian Ocean Islands” OR “Central America” OR “South America” OR “eastern Europe” OR “Transcaucasia” OR “Caribbean” OR “Pacific Islands” OR “Afghan” OR “afghani” OR “afghanistan” OR “Bangladesh” OR “bangladeshi” OR “Benin” OR “Beninese” OR “Burkina Faso” OR “Burkinabe” OR “Burundi” OR “burundian” OR “Cambodia” OR “cambodian” OR “Central African Republic” OR “central African” OR “Chad” OR “chadian” OR “Comoros” OR “comoran” OR “Congo” OR “congolese” OR “Eritrea” OR “eritrean” OR “Ethiopia” OR “ethiopian” OR “Gambia” OR “gambian” OR “Guinea” OR “guinean” OR “Haiti” OR “haitian” OR “Kenya” OR “Kenyan” OR “Korea” OR “korean” OR “Kyrgyz” OR “kyrgyzstan” OR “Liberia” OR “liberian” OR “Madagascar” OR “malagasy” OR “Malawi” OR “malawian” OR “mali” OR “malian” OR “mozambique” OR “mozambican” OR “Myanmar” OR “myanmarese” OR “burmese” OR “Nepal” OR “Nepalese” OR “Niger” OR “nigerian” OR “Rwanda” OR “rwandan” OR “Sierra Leone” OR “sierra leonean” OR “Somalia” OR “somalian” OR “Tajikistan” OR “tajik” OR “tadzhik” OR “Tanzania” OR “tanzanian” OR “Togo” OR “togolese” OR “Uganda” OR “ugandan” OR “Zimbabwe” OR “zimbabwean” OR “Angola” OR “angolan” OR “Armenia” OR “armenian” OR “Belize” OR “belizean” OR “Bhutan” OR “bhutanese” OR “Bolivia” OR “bolivian” OR “Cameroon” OR “cameroonian” OR “Cape Verde” OR “cape verdian” OR “cape verdean” OR “Côte d'Ivoire” OR “ivory coast” “ivorian” OR “Djibouti” OR “Egypt” OR “egyptian” OR “El Salvador” OR “salvadoran” OR “Fiji” OR “fijian” OR “Georgia” OR “georgian” OR “Ghana” OR “ghanaian” OR “Guatemala” OR “Guatemalan” OR “Guyana” OR “guyanese” OR “Honduras” OR “honduran” OR “Indonesia” OR “indonesian” OR “India” OR “indian” OR “Iraq” OR “iraqi” OR “Kiribati” OR “Kosovo” OR “kosovar” OR “Laos” OR “lao” OR “laotian” OR “Lesotho” OR “Marshall Islands” OR “marshallese” OR “Mauritania” OR “mauritanian” OR “Micronesia” OR “micronesian” OR “Moldova” OR “moldovan” OR “Mongolia” OR “mongolian” OR “Morocco” OR “moroccan” OR “Nicaragua” OR “nicaraguan” OR “Nigeria” OR “nigerian” OR “Pakistan” OR “pakistani” OR “Papua New Guinea” OR “papua new guinean” OR “Paraguay” OR “paraguayan” OR “Philippines” OR “filipino” OR “Samoa” OR “samoan” OR “Sao Tome and Principe” OR “São Tomé and Príncipe” OR “Senegal” OR “senegalese” OR “Solomon Islands” OR “Solomon islander” OR “Sri Lanka” OR “srilankan” OR “Sudan” OR “sudanese” OR “Swazi” OR “swaziland” OR “Syria” OR “syrian” OR “east Timor” OR “east timorese” OR “Tonga” OR “tongan” OR “Turkmenistan” OR “turkmen” OR “Tuvalu” OR “tuvaluan” OR “Ukraine” OR “ukrainian” OR “Uzbekistan” OR “uzbek” OR “Vanuatu” OR “Vietnam” OR “vietnamese” OR “West Bank”

**Table A3.** CINAHL search strategy.

#	Search strategy
1	Pharmacy services (MH “pharmacist+”) OR (MH “pharmacy+”) OR (MH “pharmacies+”) OR (TX community# N5 pharmac#) OR (TX pharmacy# N5 servic#) OR (TX pharmac# N5 store#) OR (TX pharmac# N5 shop#) OR (TX pharmac# N5 assistant#) OR (TX pharmac# N5 technician#)
2	NCDs (MH “non communicable disease+”) OR (MH “NCD+”) OR (MH “NCDs+”) OR (TX “non communicable disease*”) OR (TX “NCD*”) OR (MH “diabetes+”) OR (MH “diabetes mellitus+”) OR (TX “diabetes*”) OR (TX “diabetic*”) OR (TX “diabetes mellitus*”) OR (MH “chronic obstructive pulmonary disease+”) OR (MH “asthma+”) OR (TX “COPD*”) OR (TX “chronic obstructive pulmonary disease *”) OR (TX “asthma *”) OR (MH “breast cancer+”) OR (MH “cervical cancer+”) OR (MH “cancer screening+”) OR (TX breast# N5 cancer#) OR (TX cervical# N5 cancer#) OR (TX cancer# N5 screening#) OR (MH “lifestyle counselling+”) OR (MH “lifestyle counseling+”) OR (MH “tobacco cessation+”) OR (MH “cessation of tobacco+”) OR (MH “health education +”) OR (TX “lifestyle counselling+”) OR (TX “lifestyle counseling+”) OR (TX “tobacco cessation+”) OR (TX “cessation of tobacco+”) OR (TX “health education +”)
3	Geographical scope (MH “developing countries+”) OR (MH “medically underserved area+”) OR (TX “developing countr*”) OR (TX “medically underserved area#”) OR (TX “low income countr*”) OR (TX “middle income countr*”) OR (MH “Africa+”) OR (TX Africa#) OR (TX Caribbean) OR (MH “west indies+”) OR (TX “central America#”) OR (MH “Central America+”) OR (TX “south America#”) OR (MH “south America+”) OR (TX global) OR (TX “low resource”) OR (TX “resource poor”) OR (TX “central asia#”) OR (MH “asia, central+”) OR (TX “southeastern asia#”) OR (MH “asia, southeastern+”) OR (TX “western asia#”) OR (MH “asia, western+”) OR (TX “Indian ocean islands”) OR (MH “Indian ocean islands+”) OR (TX “eastern Europe*”) OR (MH “europe, eastern”) OR (TX Transcaucasia#) OR (TX “pacific islands”)OR (MH “pacific islands+”) OR (TX Afghan*) OR (TX Bangladesh#) OR (TX Benin*) OR (TX “Burkina Faso”) OR (TX burkinabe) OR (TX Burundi*) OR (TX Cambodia#) OR (TX “Central African”) OR (TX Chad*) OR (TX Comor*) OR (TX Congo*) OR (TX Eritrea#) OR (TX Ethiopia#) OR (TX Gambia#) OR (TX Guinea#) OR (TX Haiti*) OR (TX Kenya#) OR (TX Korea#) OR (TX Kyrgyz*) OR (TX Liberia#) OR (TX Madagascar) OR (TX malagasy) OR (TX Malawi*) OR (TX mali*) OR (TX mozambi*) OR (TX Myanmar*) OR (TX Nepal*) OR (TX Niger*) OR (TX Rwanda#) OR (TX “Sierra Leon*”) OR (TX Somalia#) OR (TX Tajik*) OR (TX tadzhik) OR (TX Tanzania#) OR (TX Togo*) OR (TX Uganda#) OR (TX Zimbabwe*) OR (TX Angola#) OR (TX Armenia#) OR (TX Belize*) OR (TX Bhutan*) OR (TX Bolivia#) OR (TX Cameroon*) OR (TX Cape Verd*) OR (TX Congo*) OR (TX “Côte d'Ivoire”) OR (TX “ivory coast”) OR (TX ivorian) OR (TX Djibouti) OR (TX Egypt#) OR (TX “El Salvador”) OR (TX “Salvadoran”) OR (TX Fiji*) OR (TX Georgia#) OR (TX Ghana*) OR (TX Guatemala#) OR (TX Guyan*) OR (TX Hondura#) OR (TX Indonesia#) OR (TX India#) OR (TX Iraq#) OR (TX Kiribati) OR (TX Kosov*) OR (TX Lao*) OR (TX Lesotho) OR (TX (“Marshall Islands”)) OR (TX marshallese) OR (TX Mauritania#) OR (TX Micronesia#) OR (TX Moldova#) OR (TX Mongolia#) OR (TX Morocc*) OR (TX Nicaragua#) OR (TX Nigeria#) OR (TX Pakistan#) OR (TX “Papua New Guinea*”) OR (TX Paraguay*) OR (TX Philippines) OR (TX Filipino) OR (TX Samoa#) OR (TX “Sao Tome*”) OR (TX Senegal*) OR (TX “Solomon Island*”) OR (TX “sri lanka#”) OR (TX Sudan*) OR (TX Swazi*) OR (TX Syria#) OR (TX Timor*) OR (TX Tonga#) OR (TX Turkmen*) OR (TX Tuvalu*) OR (TX Ukrain*) OR (TX Uzbek*) OR (TX Vanuat*) OR (TX Vietnam*) OR (TX (“West Bank”)) OR (TX Gaza) OR (TX Yemen*) OR (TX Zambia#) OR (TX Albania#) OR (TX Algeria#) OR (TX “Antigua and Barbuda”) OR (TX antiguan) OR (TX barbudan) OR (TX Argentin*) OR (TX Azerbaijani*) OR (TX Belarus*) OR (TX Bosnia#) OR (TX Botswana) OR (TX Brazil*) OR (TX Bulgaria#) OR (TX Chile*) OR (TX China) OR (TX Chinese) OR (MH “China+”) OR (TX Colombia#) OR (TX “Costa Rica#”) OR (TX Cuba#) OR (TX Dominica#) OR (TX Ecuador*) OR (TX Gabon*) OR (TX Grenad*) OR (TX Iran*) OR (TX Jamaica#) OR (TX Jordan*) OR (TX Kazakhstan#) OR (TX Latvia#) OR (TX Leban*) OR (TX Libya#) OR (TX Lithuania#) OR (TX Macedonia#) OR (TX Malaysia#) OR (TX Maldiv*) OR (TX mauriti*) OR (TX Mexic*) OR (TX Montenegr*) OR (TX Namibia#) OR (TX Palau*) OR (TX Panama*) OR (TX Peru*) OR (TX Romania#) OR (TX Russia#) OR (TX Serbia#) OR (TX Seychell*) OR (TX “South Africa#”) OR (TX “Saint Kitts”) OR (TX “Saint Lucia”) OR (TX “Saint Vincent”) OR (TX Suriname#) OR (TX Thai*) OR (TX Tunisia#) OR (TX Turk*) OR (TX Uruguay*) OR (TX Venezuala#)
